# Differential distribution of a SINE element in the *Entamoeba histolytica *and *Entamoeba dispar *genomes: Role of the LINE-encoded endonuclease

**DOI:** 10.1186/1471-2164-12-267

**Published:** 2011-05-25

**Authors:** Vandana Kumari, Rahul Sharma, Vijay P Yadav, Abhishek K Gupta, Alok Bhattacharya, Sudha Bhattacharya

**Affiliations:** 1 School of Environmental Sciences, Jawaharlal Nehru University, New Delhi 110067, India; 2 School of Life Sciences, Jawaharlal Nehru University, New Delhi 110067, India

## Abstract

**Background:**

*Entamoeba histolytica *and *Entamoeba dispar *are closely related protistan parasites but while *E. histolytica *can be invasive, *E. dispar *is completely non pathogenic. Transposable elements constitute a significant portion of the genome in these species; there being three families of LINEs and SINEs. These elements can profoundly influence the expression of neighboring genes. Thus their genomic location can have important phenotypic consequences. A genome-wide comparison of the location of these elements in the *E. histolytica *and *E. dispar *genomes has not been carried out. It is also not known whether the retrotransposition machinery works similarly in both species. The present study was undertaken to address these issues.

**Results:**

Here we extracted all genomic occurrences of full-length copies of EhSINE1 in the *E. histolytica *genome and matched them with the homologous regions in *E. dispar*, and vice versa, wherever it was possible to establish synteny. We found that only about 20% of syntenic sites were occupied by SINE1 in both species. We checked whether the different genomic location in the two species was due to differences in the activity of the LINE-encoded endonuclease which is required for nicking the target site. We found that the endonucleases of both species were essentially very similar, both in their kinetic properties and in their substrate sequence specificity. Hence the differential distribution of SINEs in these species is not likely to be influenced by the endonuclease. Further we found that the physical properties of the DNA sequences adjoining the insertion sites were similar in both species.

**Conclusions:**

Our data shows that the basic retrotransposition machinery is conserved in these sibling species. SINEs may indeed have occupied all of the insertion sites in the genome of the common ancestor of *E. histolytica *and *E. dispar *but these may have been subsequently lost from some locations. Alternatively, SINE expansion took place after the divergence of the two species. The absence of SINE1 in 80% of syntenic loci could affect the phenotype of the two species, including their pathogenic properties, which needs to be explored.

## Background

Transposable elements are found in the genomes of almost all organisms, and are of ancient origin. Their ability to insert into new genomic locations makes them potent agents of phenotypic change, including various known pathologies [1-3]. Transposons are also found in parasites, for example the human enteric pathogen, *Entamoeba histolytica*, a unicellular eukaryote contains three families of the autonomous non long terminal repeat (LTR) retrotransposon called EhLINE and its nonautonomous partner EhSINE [4-8]. The genome of the morphologically indistinguishable sibling species *Entamoeba dispar*, (which resides in the human colon but is nonpathogenic), also contains three families of EdLINEs and their partner EdSINEs [6,7,9]. It would be of interest to know whether these retrotransposons have any influence on pathogenesis-related gene expression.

LINEs and SINEs are known to influence the expression of neighboring genes by a variety of mechanisms, for example by providing alternative promoters, splicing and polyadenylation sites and by heterochromatinization [10-13]. For this reason it is important to investigate whether these elements are located at syntenic positions in the *E. histolytica *and *E. dispar *genomes. Earlier investigations with a limited number of genomic loci showed that the sites occupied by EhSINE1 in the *E. histolytica *genome were empty at homologous regions in *E. dispar *[14] and conversely the sites occupied by EdSINE1 in the *E. dispar *genome were empty in *E. histolytica *[9]. Although an exhaustive genome-wide survey of LINEs and SINEs in *Entamoeba *species has been reported [6], a genome-wide comparison of the occupancy of these elements at syntenic loci in *E. histolytica *and *E. dispar *has not been carried out. Here we present results of a genome-wide comparison of EhSINE1 and EdSINE1 in the two genomes. In addition we address the question whether the differences in genomic locations of retrotransposons in these two organisms could be due to inherent differences in the retrotransposition machinery, particularly in the properties of the LINE-encoded endonuclease. Target primed reverse transcription is the mechanism by which non-LTR retrotransposons insert in the genome [15]. Since retrotransposition is initiated by the element-encoded endonuclease (EN) making a nick at the bottom strand of the site of insertion, an important determinant of target site specificity could be the preferred nucleotide sequences recognized by the EN. We have earlier shown that the EhLINE1-encoded EN (Eh EN) nicks preferentially at the consensus sequence 5'-GTATT-3', between A-T and T-T. Here we have investigated whether the endonuclease domain encoded by EdLINE1 (Ed EN) has a different target site specificity which could account for the lack of synteny in the location of SINEs in the two genomes.

## Methods

### Comparative Analysis of *E. histolytica *and *E. dispar *with respect to the occupancy of SINE1 elements

Since the *Entamoeba *genome has not yet been assembled completely for any species, we have done all of our comparative analysis on the basis of SINE1 elements located on the scaffolds. The genome sequence of *E. histolytica *having 1529 scaffolds and *E. dispar *having 12258 scaffolds was downloaded [NCBI:AAFB00000000, NCBI:AANV00000000]. A total of 393 EhSINE1 and 302 EdSINE1 elements having lengths greater than 450 bp were located. The EhSINE1 sequences were from Huntley et al [4] and the EdSINE1 sequences were from the feature table file of *E. dispar *(updated on Dec. 8, 2008). For genes flanking the EhSINE1 sequences the feature table file of *E. histolytica *(updated on April 17, 2008) was used. These were downloaded from NCBI and the genes upstream and downstream of SINE1 elements in each of the species were located using perl coding. Finally the orthologues of these genes were searched in the other species using BLAST [16]. The syntenic loci were checked to see whether SINE1 was present there in both species. To check the homology at Scaffold level we used GATA, a graphic alignment tool for comparative sequence analysis [17]. Syntenic loci were further examined to check for the presence of SINE in both species.

DNA sequence features of the SINE1 insertion sites were computed by extracting flanking 80 bp sequences (40 bp upstream and downstream) from the respective genomic sites in *E. histolytica *and *E. dispar*, using perl scripting. "DNA SCANNER" was used as previously described [18] to calculate the DNA parameters, which include T rule, bendability, propeller twist, stacking energy, duplex stability, DNA denaturation, protein-induced deformability, nucleosomal positioning and bending energy [18].

### Construction and Cloning of Ed EN

The consensus amino acid sequences of the *E. dispar *and *E. histolytica *endonuclease domains, Ed EN and Eh EN respectively were compared. A total of 23 amino acid positions were different of which 12 amino acid residues were changed in the Eh EN sequence [19] and 11 amino acid residues (synonymous) were left unchanged to obtain the Ed EN sequence. The desired mutations were introduced either by means of overlapping PCR (Additional file 1 Figure S1) or by site-directed mutagenesis. The primer pairs used are listed (Additional file 2 Table S1). The Ed EN sequence thus obtained (782 bp) was cloned into the *Eco*RI-*Not*I site of pET30(b) vector (Novagen) to yield the pET-Ed-EN construct.

### Expression and purification of recombinant Ed EN

This was done essentially as described for Eh EN [19]. The pET-Ed-EN construct was transformed in *Escherichia coli *BL-21 (DE3). Cells were grown in 200 ml of Luria Broth at 30°C to OD_600 _of 0.6. For induction of hexa-His-tagged Ed EN, IPTG was used to a final concentration of 0.5 mM and the cells were further incubated for 2 hour. The protein was purified by Ni^2+^-nitrilotriacetic acid-agarose (Qiagen) affinity chromatography and eluted with 250 mM of imidazole. The eluted fractions were checked for the protein by resolving on a 12% SDS PAGE; these fractions were pooled, dialyzed and stored at -20°C. The enzyme activity of Ed EN was measured under the same conditions used for Eh EN, i.e. pH 7.0, at 37°C, and at Mg^2+ ^and NaCl concentrations of 10 and 100 mM respectively [20].

### Western blotting

The whole cell lysate of induced and uninduced BL-21 (DE3) cells was resolved on 12% SDS PAGE and transferred to PVDF membrane by semidry transfer method according to manufacturer's instructions (Bio-Rad). The membrane was blocked overnight with 5% skimmed milk in PBS-T (Phosphate buffer saline with 0.1% Tween 20) and subsequently incubated with anti-His antibody or anti-Eh EN antibody for 1 hour followed by three times washing with PBS-T. The membrane was further incubated with horseradish peroxidase-conjugated secondary antibody and again washed with PBS-T thrice. The protein was detected by the Chemiluminescent HRP Substrate (Millipore).

### Nicking assay of radiolabeled 176 bp substrate

The 176 bp DNA fragment was radiolabeled as described earlier [19]. The labeled DNA was resolved on 6% native gel, the band was visualized by ethidium bromide staining, excised from the gel and eluted by crush and soak method [21]. The nicking assay was carried out as described for Eh EN and products were resolved by denaturing electrophoresis on 6-8% polyacrylamide gels containing 7 M Urea [19]. The gels were dried and autoradiographed in the PhosphorImager (Fujifilm).

### Nicking assay of pBS DNA

Supercoiled pBluescript (pBS) plasmid DNA was purified by plasmid purification kit (Qiagen). 2 nM of purified Ed EN was incubated with 2-75 nM of supercoiled pBS DNA. The reaction was performed under the conditions employed for Eh EN [20], at 37°C for 8 minutes and was stopped with 25 mM EDTA. Products were separated on 0.8% agarose gel containing 0.5 μg of ethidium bromide/ml. Under this condition the supercoiled DNA migrated fastest, followed by linear and open circular form. The intensity of supercoiled DNA band was measured as a function of time, which gave the measure of the disappearance of supercoiled DNA. Quantification was done by densitometry; the kinetic constants V_max_, K_m _and k_cat _were determined as described [22]. The data obtained were the average of three independent determinations.

## Results and Discussion

### Comparative analysis of EhSINE1-containing regions of *E. histolytica *genome and syntenic regions of *E. dispar*

**Figure 1:**
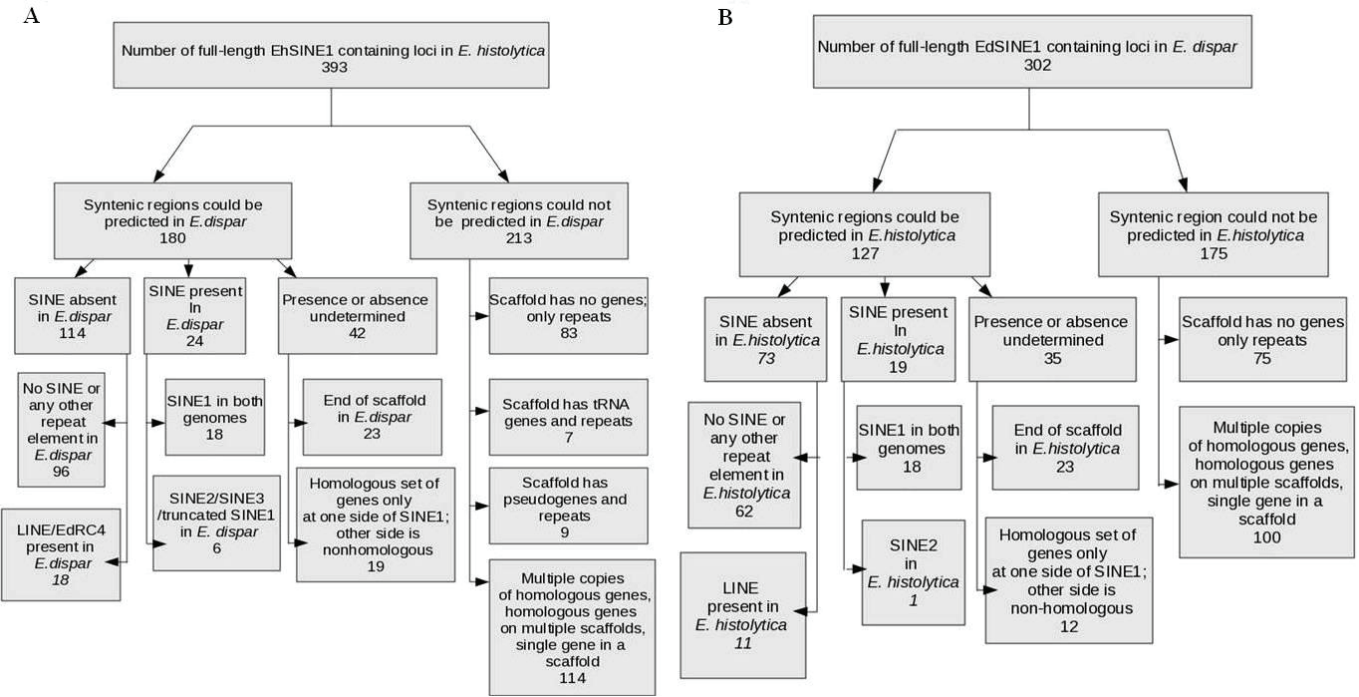
**Summary of the comparative analysis of genomic locations of SINE1 copies in *E. histolytica *and *E. dispar***. (A). Comparison of EhSINE1-occupied regions in *E. histolytica *genome with syntenic regions of *E. dispar*. (B). Comparison of EdSINE1-occupied regions in *E. dispar *genome with syntenic regions of *E. histolytica*.

**Figure 2:**
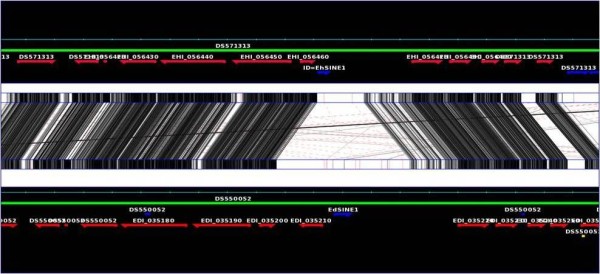
**Graphical representation of a syntenic region where SINE1 is present in both *E. histolytica *and *E. dispar***. Top and bottom green lines mark the genomes of *E. histolytica *and *E. dispar *respectively. The *E. histolytica *and *E. dispar *genes are marked by red arrows, showing the gene orientation. Shaded black lines showing the identical region within *E. histolytica *and *E. diapar*. Darkness of the shade is proportional to the % identity, with white being the least conserved. Blue arrows show the repeat elements (EhSINE1 and EdSINE1).

A total of 393 full-length SINE1 elements (length > 450 bp) were identified in *E. histolytica *by genome sequence analysis. Syntenic regions corresponding to each of the EhSINE1-containing loci were located in *E. dispar*. To score for synteny entire scaffolds were matched in the two species using the program GATA. In 88% of cases where synteny was found, the syntenic regions matched throughout the scaffold, while in the rest synteny was not visible in some patches. The presence or absence of any of the EdSINEs (EdSINE1, 2, 3) was determined at syntenic loci of *E. dispar*. The results are summarized in Figure 1A. Of the 393 EhSINE1-containing loci of *E. histolytica*, syntenic regions in *E. dispar *could be predicted with certainty for 180 loci. Only these loci were included for further study - thus removing the contribution of differential sequence coverage in our comparative analysis. Loci not represented in both species due to differences in genome coverage, or difficulty in alignment, have not been included. In addition, as stated above, the synteny stretched for the entire length of the scaffold. Of these 180 loci, SINEs were absent in *E. dispar *at 114 loci, were present at 24 loci and their presence or absence could not be determined at 42 loci. Amongst the 114 loci where SINEs were absent, no repeat element of any type was found at 96 loci (representative example shown in Additional file 3 Figure S2), while LINE or EdRC4 sequences were found at 18 loci. Amongst the 24 loci that contained a SINE, 18 had EdSINE1 (one representative example is shown in Figure 2 and the rest in Additional file 4 Figure S3-S19), one had a truncated copy of EdSINE1, and five had EdSINE2 and/or EdSINE3. Amongst the 42 loci where presence or absence of SINE could not be established, in 23 cases the scaffold ended within the locus in *E. dispar*, and in 19 cases the homology was restricted to genes on one side of the SINE while there was no homology on the other side (may be due to deletions/inversions/rearrangements).

For 213 EhSINE1-containing loci of *E. histolytica*, syntenic loci could not be found in *E. dispar *for the following reasons. In many of these cases the scaffolds containing these loci were composed entirely of repeats (83 loci), tRNA genes and repeats (7 loci) or pseudogenes and repeats (9 loci). In 114 cases synteny could not be determined either because there were multiple copies of homologous genes, or homologous genes were located on multiple scaffolds, or there was a single gene in the scaffold.

### Comparative analysis of EdSINE1-containing regions of *E. dispar *genome and syntenic regions of *E. histolytica*

A total of 302 full-length SINE1 elements (length greater than 450 bp) were identified in *E. dispar *by genome sequence analysis. Syntenic regions corresponding to each of the EdSINE1-containing loci were located in *E. histolytica *and the presence or absence of any of the EhSINEs (EhSINE1, 2, 3) was determined as described above. Of the 302 loci, syntenic regions could be predicted with certainty for 127 loci (Figure 1B). Of these, SINEs were absent in *E. histolytica *at 73 loci, were present at 19 loci and their presence or absence could not be determined at 35 loci. Amongst the 73 loci where SINEs were absent, no repeat element of any type was found at 62 loci (Additional file 5 Figure S20), while LINE sequences were found at 11 loci. Amongst the 19 loci that contained a SINE, 18 had EhSINE1 (as scored above) while 1 had EhSINE2. Amongst the 35 loci where presence or absence of SINE could not be established, in 23 cases the scaffold ended within the locus in *E. histolytica*, and in 12 cases the homology was restricted to genes on one side of the SINE while there was no homology on the other side.

For 175 EdSINE1-containing loci, syntenic loci could not be found in *E. histolytica *for the reasons mentioned in the previous section. In 75 cases the *E. dispar *loci were composed entirely of repeats while in 100 cases synteny could not be determined either because there were multiple copies of homologous genes, or homologous genes were located on multiple scaffolds, or there was a single gene in the scaffold.

### Sequence alignment of syntenic loci

**Figure 3:**
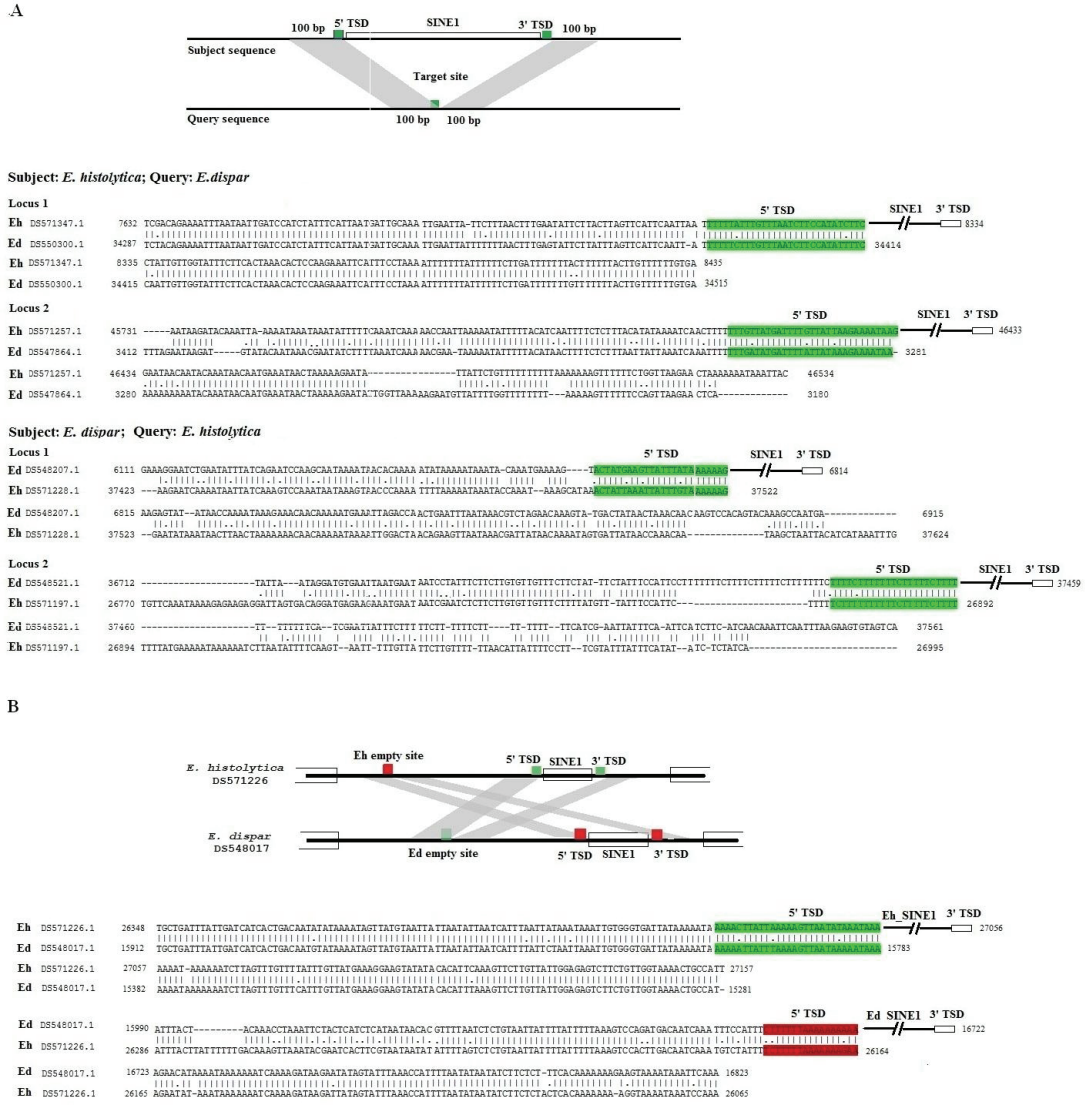
**Sequence alignment of syntenic intergenic regions**. (A) SINE1 is absent in one of the species; (B) SINE1 is present in both species. The scaffold number and nucleotide positions are indicated. TSD, target site duplication.

Figure 3 shows actual sequence alignments of a few selected syntenic loci where SINE1 is found in *E. histolytica *but missing in *E. dispar*, and vice versa. As is evident, in each case the element is flanked by TSDs. Only one copy of the TSD is found at the syntenic locus of the species where the SINE is missing. The surrounding sequences show the sequence similarity expected of intergenic regions of the two species (80-90%). One example is shown of an intergenic region where SINE1 is present in both species. Although SINE1 is located in the same intergenic region, the actual point of insertion is not the same and consequently the TSD sequences are different (Figure 3B). This was the typical pattern seen in other loci of this type where SINEs were located in the same intergenic regions in both species.

From the above data it is clear that only in about 20% of cases where presence or absence of SINE1 could be established at syntenic loci, are SINE elements located in the same intergenic region (although at different insertion points) in both species. In >80% of these loci SINE1 was not found at the same location in both species. Since the elements in the two species have a common lineage and are closely related, what possible factors might account for these differences? According to the Target primed Reverse Transcription model, retrotransposition is initiated by the LINE-encoded Endonuclease (EN) nicking the bottom strand of the target site [15]. Hence it is reasonable to believe that the sequences preferentially nicked by the EN could be the preferred insertion sites of the retrotransposon, and the behavior of EN might influence the choice of target site of a non LTR retrotransposon. Since the Eh EN and Ed EN differ from each other at many amino acid positions (as shown below), it is possible that the two enzymes may have evolved different recognition specificities. To establish this we studied the properties of the EdLINE1-encoded EN and compared it with EhLINE1-encoded EN.

### Cloning and expression of the EdLINE1 endonuclease (Ed EN) polypeptide

**Figure 4:**
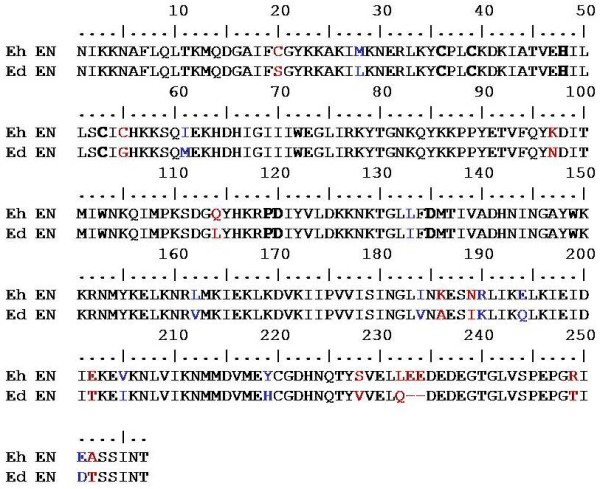
**Amino acid sequence alignment of consensus Eh LINE1 EN and Ed LINE1 EN**. The conserved motifs CCHC and PD (X) _10-14 _D (shown in bold) are present both in Eh EN and Ed EN. Amino acid residues which were mutated to construct Ed EN are shown in red and the synonymous amino acid residues left unchanged are shown in blue.

**Figure 5:**
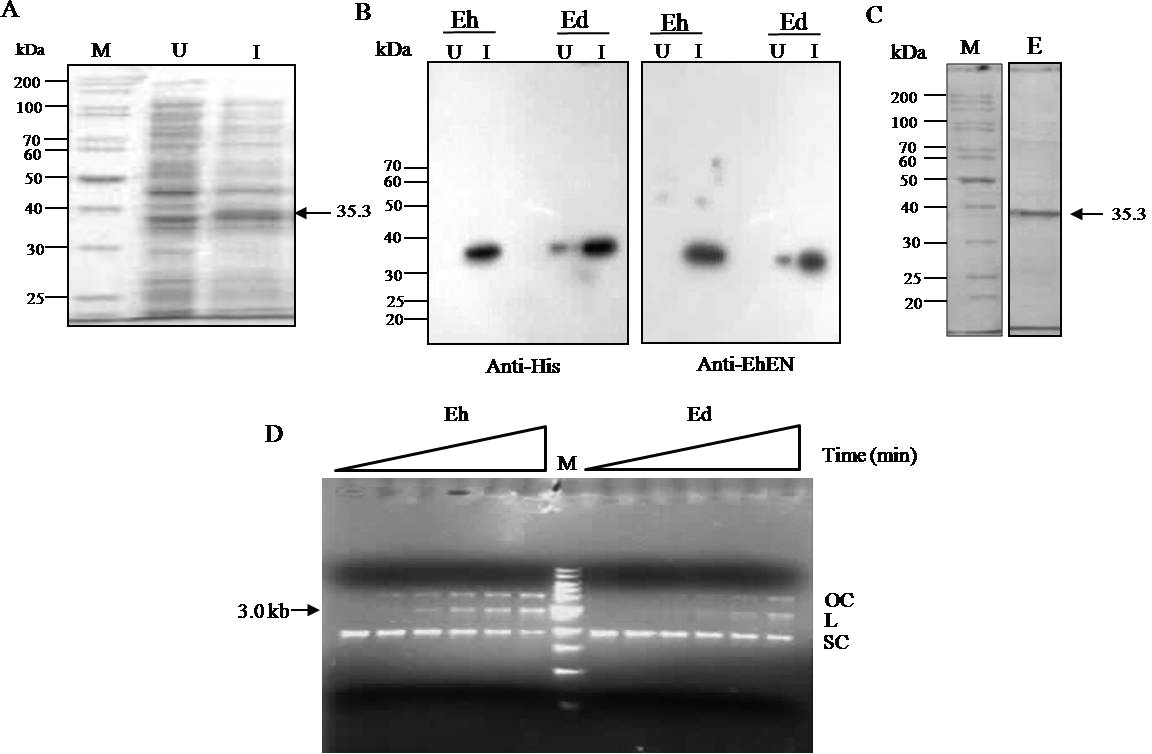
**Purification of Ed EN and cleavage activity on pBS supercoiled DNA**. (A) The Ed EN protein was expressed in the *E. coli *expression vector pET30(b) by inducing with IPTG. Expression was checked by separating uninduced (U) and induced (I) whole cell lysate on 12% denaturing polyacrylamide gel. The expected size (35.3 kDa) is marked. (B) Western blot analysis with anti-his tag and anti-Eh EN antibody was performed using whole cell lysate from above figure. Eh EN was loaded for comparison in the 'Eh' lanes. (C) Ed EN was purified through Ni-NTA affinity chromatography and checked by separating on 12% denaturing polyacrylamide gel. (D) The cleavage activity of Ed EN was checked by incubating purified protein with pBS supercoiled DNA at 37°C under conditions earlier used for Eh EN. The figure shows agarose gel picture of time course (0, 5, 10, 15, 30 and 60 min) of incubation with Eh EN and Ed EN. The position of 3.0 kb band corresponding with linear pBS DNA is marked. OC, open circular; L, linear; SC, supercoiled.

To obtain the Ed EN coding sequence we used the Eh EN sequence (already cloned in our lab) as a starting point. Ed EN differs from Eh EN in 23 amino acid positions (Figure 4). The Eh EN sequence was mutated in these positions (as described in Methods) to obtain the Ed EN coding sequence. This 782-bp *Eco*RI-*Not1 *fragment was cloned in the *E. coli *expression vector pET30b. The expressed protein contained His-tag, and together with other vector sequences at the amino terminus, it was 307 amino acids long, with an expected molecular mass of 35.3 kDa (Figure 5A). It was purified by nickel-agarose chromatography, and its identity was confirmed by using an anti-His tag antibody and anti-Eh EN antibody (Figure 5B). The Ed EN protein could nick a nonspecific substrate, pBS. Like the previously reported activity of Eh EN [19] supercoiled pBS DNA was efficiently nicked by the purified Ed EN protein to yield open circular and linear DNAs. The presence of discrete bands corresponding to open circular and linear forms shows that the enzyme makes predominantly single-strand nicks and not double-strand breaks (Figure 5D).

### Kinetics of the Ed EN-catalyzed reaction with pBS supercoiled DNA substrate under steady-state conditions

**Figure 6:**
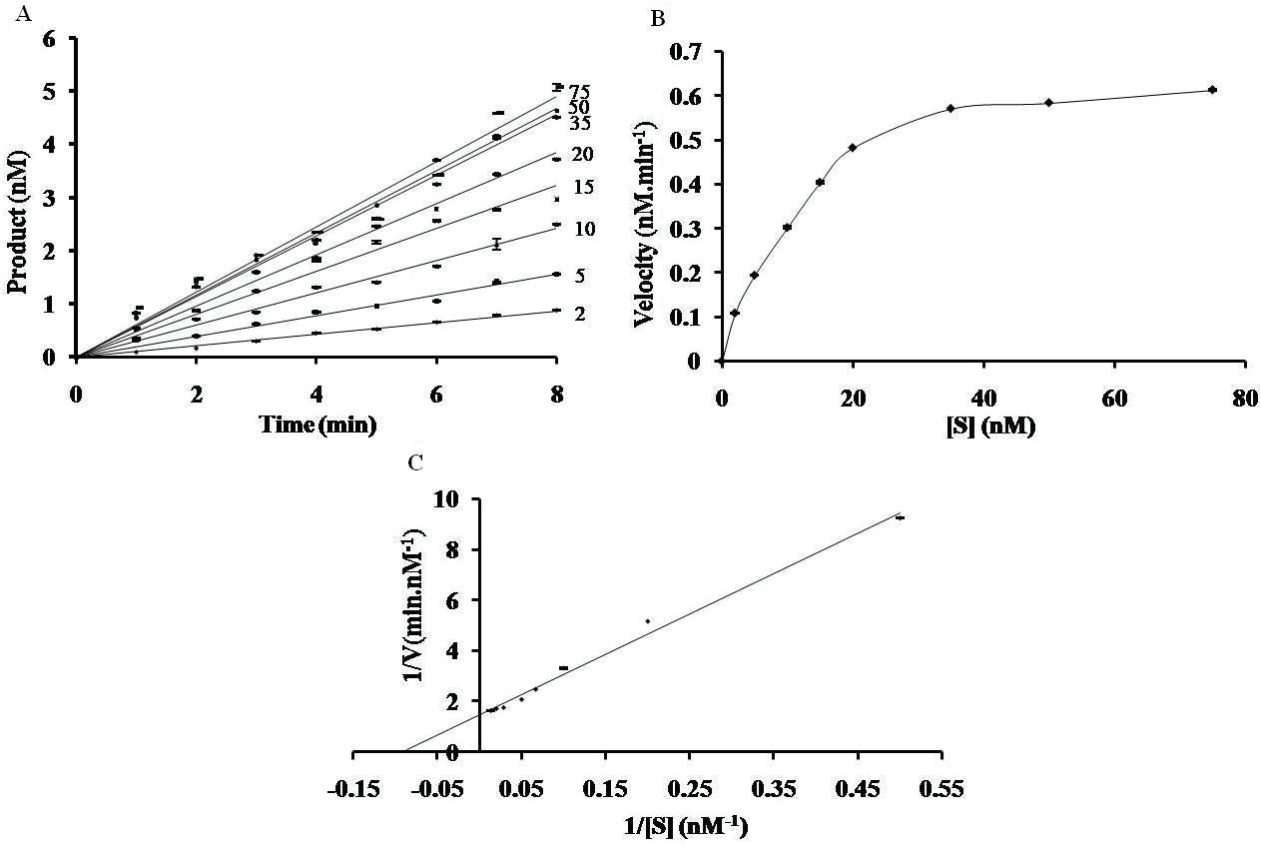
**Kinetics of cleavage of supercoiled pBS DNA by Ed EN**. (A) Steady-state kinetics. Assays were carried out at 37°C with 2 nM Ed EN in a reaction mixture containing increasing concentrations (2-75 nM) of pBS DNA. Aliquots were withdrawn at different time-points (0-8 min) during the reaction and assayed by electrophoresis through 0.8% agarose. The concentration of the supercoiled form of pBS DNA at each time-point was quantified as described in the Materials and Methods. The disappearance of the supercoiled form of pBS DNA with time was plotted for the indicated concentration of substrate (nM) and the slopes thus obtained were taken as initial velocity at corresponding substrate concentrations. (B) and (C) DNA cleavage as a function of substrate concentration. Initial reaction velocities, obtained as described above, were plotted as a function of substrate concentration. A Lineweaver-Burk plot (C) was used to calculate the kinetic parameters K_m _and k_cat_. The data are expressed as the average of three independent determinations, as mentioned in the Results and Discussion, and the standard deviation is indicated as error bars (± SD).

To determine the kinetics under steady-state conditions, reactions were carried out with the enzyme at a concentration of 2 nM and with pBS DNA at a concentration of 2-75 nM (Figure 6). The disappearance of supercoiled DNA was determined by densitometric scanning as described for Eh EN [20]. As mentioned in Methods, all time-course results were the average of at least three independent determinations. The variation observed at each time point was <4.7% of the mean value (0.09-5.1). Although the variation in values of each data point was in the range of 4% in three replicates, the slopes for each set showed lesser variation (up to 1.0%). Kinetic parameters (K_m _and k_cat_) were calculated from a Lineweaver-Burk plot (Figure 6C). K_m _for pBS DNA was calculated to be 1.086 ± 0.009 × 10^-8 ^M. The catalytic constant, k_cat_, (V_max _⁄ [E]) was determined to be 5.67 ± 0.027 × 10^-3 ^sec^-1^. These values were comparable to the K_m _(2.6 ± 0.018 × 10^-8 ^M) and k_cat _(1.6 ± 0.01 10^-2 ^sec^-1^) of Eh EN [20] and the K_m _was comparable with the low K_m _values (0.5-17 nM) of restriction endonucleases determined with different DNA substrates under different conditions of buffer and temperature [22]. Furthermore, the turnover number (k_cat_) of the enzyme was in the lower range of that reported for restriction endonucleases [(1.6-16.6) x10^-2 ^sec^-1^] [22]. The low turnover number of a retrotransposon-encoded endonuclease may have a significant role in limiting the rate of retrotransposition events in the genome. From the above data we infer that the kinetic parameters of Ed EN are not significantly different from Eh EN.

### Nicking site sequence preference of Ed EN

**Figure 7:**
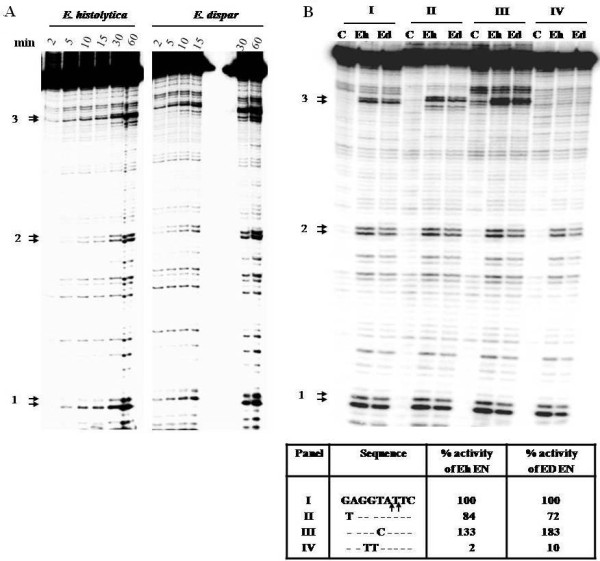
**Comparative nicking profile of Eh EN and Ed EN with 176 bp substrate containing an unoccupied insertion site of EhSINE1**. (A) End labeled 176 bp substrate was incubated with each enzyme for the indicated time. The reaction products were separated through 8% polycarylamide gel containing 7 M urea as mentioned in Materials and Methods. The three major nicking sites seen with Eh EN are marked. (B) Nicking activity of Eh EN and Ed EN on substrates mutated at site #3 (sequences flanking the nicking sites are shown in the box). Substrate I is wild type and mutations in substrates II-IV are indicated. Arrows in sequence I indicate nicking sites. Lane C in each panel indicates 0 min. incubation. Each substrate was incubated with enzyme for 60 min. The ratio of band intensity of sie#3/site#2 for each mutated substrate was calculated and compared with control substrate to obtain the percentage activity.

We had earlier shown that Eh EN preferentially nicked a 176 bp fragment from *E. histolytica *precisely at the site where a SINE1 element was known to insert in this region of the genome [19]. To test whether Ed EN had a similar sequence preference as Eh EN, the same 176 bp fragment was incubated with Ed EN. The nicking pattern, determined for the bottom strand, was exactly the same as that obtained with Eh EN. Three nicking hot spots were obtained, of which site #3 corresponded with the exact site of insertion of Eh SINE1 *in vivo *(Figure 7A). The sequences important for target site recognition, as determined for Eh EN [18], were tested for Ed EN by altering the sequences immediately surrounding the nicked site #3. Transition mutations were introduced using oligonucleotides with the appropriately altered sequence to PCR amplify a 117 bp fragment from the 176 bp template (position 60 to 176). The DNA sequences thus obtained contained a normal site #2 and a mutated site #3. The activity of Ed EN on the mutated site #3 was quantitated using site #2 as an internal control. The results showed that changing the GG nucleotides (on top strand, upstream of the nick) to TT decreased the activity to 10% for Ed EN (compared with 2% for Eh EN) and changing the T nucleotide upstream of the nick to C increased the activity to 183% for Ed EN (compared with 133% for Eh EN) (Figure 7B). These results show that both Ed EN and Eh EN are very similar in their target site specificity. Since the endonuclease domain of ORF2 was used in these studies, the possibility of a complete ORF2 protein displaying a different specificity *in vivo *cannot, however, be entirely ruled out.

### DNA structural features of *E. dispar *and *E. histolytica *SINE1 insertion sites

**Figure 8:**
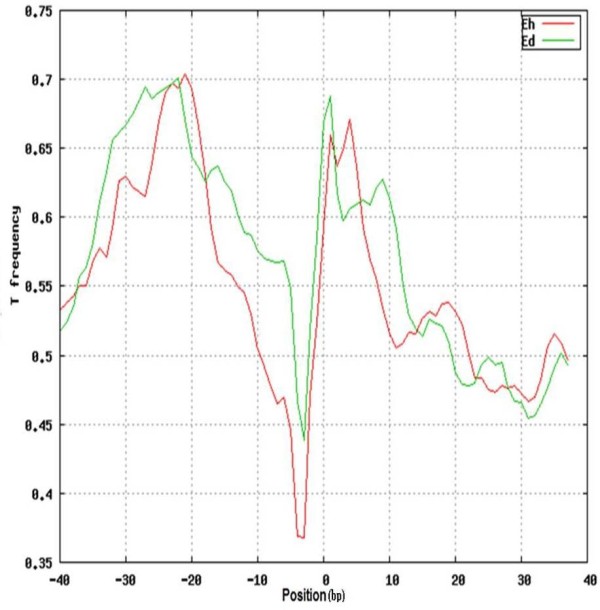
**T content profile**. Profile shows the frequency of T residues flanking the SINE1 insertion sites. Details are provided in Materials and Methods. Both *E. histolytica *(red) and *E. dispar *(green) show high T content at -22 position.

We have earlier shown that sequence-dependent DNA structural features may play an important role in site selection by SINE elements in *E. histolytica *[18]. Here we have analyzed the same features in *E. dispar *to see if the SINE insertion sites share these properties with *E. histolytica*. The parameters checked are listed in 'Methods'. In general majority of the features showed similar pattern in both the species except for DNA denaturation energy and free energy profile. Our previous study had shown that the insertion sites in *E. histolytica *are T-enriched and the content profile showed a significant peak at -22 bp relative to the insertion site. Similar profile was also observed for *E. dispar *with a peak at -22 bp (Figure 8). Statistical analysis by Mann-Whitney test on the difference of the average T content of insertion sites for *E. histolytica *and *E. dispar *suggested that there is no significant difference between the two. Similar results were obtained when other DNA-based structural parameters were determined. Thus the SINE1-occupied sites in *E. histolytica *and *E. dispar *share the same structural features.

We checked whether the intergenic regions at loci where SINEs were found in both genomes shared greater sequence similarity compared with loci where SINEs did not occur in both genomes. However this was not found to be the case. Intergenic regions in both sets of loci showed overall sequence similarity in the range of 75-90%. *E. histolytica *and *E. dispar *are very closely related sibling species [23] which were in fact classified as a single species until they were re-described as two separate species [24]. Their close relationship is also evident from phylogeny based on LINE-derived RT sequences. This analysis showed that all three families of LINEs and SINEs already existed in the common ancestor before *E. histolytica *and *E. dispar *separated into two distinct species [6]. The great similarity between the two ENs of *E. histolytica *and *E. dispar*, as found in this study, again shows that the basic retrotransposition machinery is highly conserved in these sibling species. It is therefore possible that SINEs may indeed have occupied all of the potential insertion sites in the genome of the common ancestor of *E. histolytica *and *E. dispar *but many of the inserted elements may have been preferentially lost in each genome as the two species diverged from each other. Indeed the differential loss of retrotransposons from specific loci might have contributed to speciation [25,26]. On the other hand it may be possible that SINE expansion took place after the divergence of the two species, and only a sub set of the potential insertion sites in the *E. histolytica *and *E. dispar *genomes are currently occupied. In that case one may expect that each of these extant genomes may possess a large number of 'empty' sites where SINEs could potentially insert in future. A hallmark of retrotransposition is the appearance of target site duplication (TSD) following the insertion of a new element. In syntenic loci an unoccupied site is expected to have one copy of the TSD which is duplicated in the occupied site. We checked for TSD sequences in *E. dispar *unoccupied sites corresponding to the syntenic *E. histolytica *occupied sites. We randomly picked 75 loci of *E. histolytica*, where SINE1 is absent in *E. dispar *and looked for matches with the TSD at each locus. At 19 loci we found very good match with the TSD sequence (matched length greater than 15 bp, and sequence identity greater than 85%). The occurrence of close matches of TSD sequences in the syntenic loci of *E. dispar *suggests that potential empty sites may exist where future retrotransposition events could take place.

## Conclusions

Our data show that the LINE-encoded endonucleases, Eh EN and Ed EN are essentially very similar, both in their kinetic properties and in their substrate sequence specificity. The DNA structural features of SINE-occupied sites in *E. histolytica *and *E. dispar *are also similar. However the elements do not insert at the same sites in the two species. Even in the 20% cases where the elements are located in the same intergenic regions in the two genomes the exact point of insertion is not the same. It is possible that, despite the Eh EN and Ed EN being very similar, the complete retrotransposition machinery consisting of the ribonucleoprotein assembly of LINE-encoded ORF1, ORF2 and the SINE transcript might function differently in the two species, thus leading to the observed differences. Since the elements are very closely related, these differences are likely to be the result of subtle changes that got established after the divergence of the two species. An experimental test of these functional changes requires the complete assembly of ORF1 and ORF2 of EdLINE1. On the other hand the possibility exists that the retrotransposition machineries in the two species are in fact identical and the observed differences are due to the stochastic nature of the insertion process. The number of potential insertion sites of these elements is likely to be large since they do not insert at specific sequences. The only known specificity of the process is that the elements in both *E. histolytica *and *E. dispar *insert only in intergenic regions and not within genes. Within intergenic regions they insert near T-rich stretches [18,19]. As discussed above, in this scenario one would expect to find a large number of 'empty' sites in each genome which could be targets of future insertions. This needs to be experimentally tested. A further possibility is that common insertions did indeed occur in the two genomes, but these were subsequently lost in a differential manner due to selection pressure. If the loss of SINE copies from specific locations conferred a growth advantage to either species, these elements could well have shaped the physiological evolution of the two species, including their virulence properties.

Whatever may have been the mechanisms and processes that determined the positioning of SINEs, the resultant effect is that SINEs occupy different intergenic locations in the two genomes. It is well recognized that LINEs and SINEs can modulate the expression of genes in their vicinity by providing alternative promoters, splicing and polyadenylation sites and by heterochromatinization [10-13]. The absence of SINE1 in >80% of syntenic loci in the extant genomes of *E. histolytica *and *E. dispar *could result in differential expression of genes at these loci. This could profoundly influence the phenotype of the two species, which needs to be explored. Recently Lorenzi et al. [27] in their reannotation and analysis of the *E. histolytica *genome have listed a large number of protein families showing high association with repetitive elements. Though the top three families which are associated with TEs 100% of the time are hypothetical proteins, important known protein families are also listed. These include gal/gal Nac lectin, hsp70 BspA-like surface protein family and AIG family associated with resistance to bacteria. Further analysis of a similar nature with *E. dispar *genome will give interesting information on the possible contribution of TEs in regulating the expression of important genes that may influence pathogenesis.

## Authors' contributions

SB proposed and designed the research, drafted the final version of the manuscript, AB designed and analyzed the computational work. VK performed the experiments regarding cloning, expression, purification and assay of Ed EN. RS performed the computational work. VPY helped in performing the enzyme kinetics of Ed EN, AKG helped in the cloning of Ed EN. All authors have participated in preparing the manuscript. All authors have read and approved the final manuscript.

## Supplementary Material

Additional file 1 **Figure S1**. Schematic representation of overlapping PCRs for construction of Ed EN. Sixteen sets of primers incorporating the desired mutations were designed. PCR was done using pET-Eh-EN construct as template. The 782 bp fragment containing Ed EN domain was cloned in pET30(b) vector at the *Eco*RI-*Not*I site. Click here for file

Additional file 2 **Table S1**. Primer sets used for the construction of Ed EN. Click here for file

Additional file 3 **Figure S2**. Graphical representation of the syntenic region where SINE1 is present in *E. histolytica *but absent in *E. dispar*. For color code and arrows refer to figure S1-S17 Click here for file

Additional file 4 **Figure S3-S19**. Graphical representation of the syntenic region where SINE1 is present in both *E. histolytica *and *E. dispar*. Top and bottom green lines are showing the genomes of *E. histolytica *and *E. dispar *respectively. Red arrows show the Eh and Ed genes, and the gene orientation. Shaded black or Red (connecting) lines show the identical region within *E. histolytica *and *E. dispar*. Darkness of the shade is proportional to the % Identity. Blue arrows show the repeat elements. SINE1 has been shown above the marked Yellow dot in both *E. hitolytica *and *E. dispar *in the syntenic region. Click here for file

Additional file 5 **Figure S20**. Graphical representation of syntenic region where SINE1 is present in *E. dispar *but absent in *E. histolytica*. For color code and arrows refer to figure S1-S17. Click here for file
