# Supplementary figures and images for: Differential distribution of a SINE element in the Entamoeba histolytica and Entamoeba dispar genomes: Role of the LINE-encoded endonuclease
Source: BMC Genomics. 2011 May 25;12:267. doi: 10.1186/1471-2164-12-267 (PMC3118788; doi:10.1186/1471-2164-12-267)

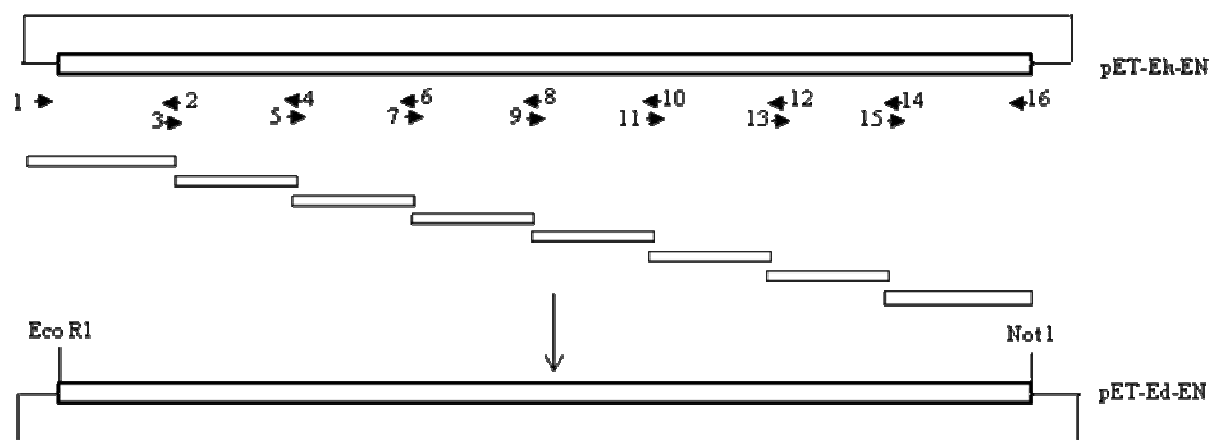

Figure. S1.

Supplement: Additional file 1 — Figure S1. Schematic representation of overlapping PCRs for construction of Ed EN. Sixteen sets of primers incorporating the desired mutations were designed. PCR was done using pET-Eh-EN construct as template. The 782 bp fragment containing Ed EN domain was cloned in pET30(b) vector at the EcoRI-NotI site. [file 1471-2164-12-267-S1.PDF]

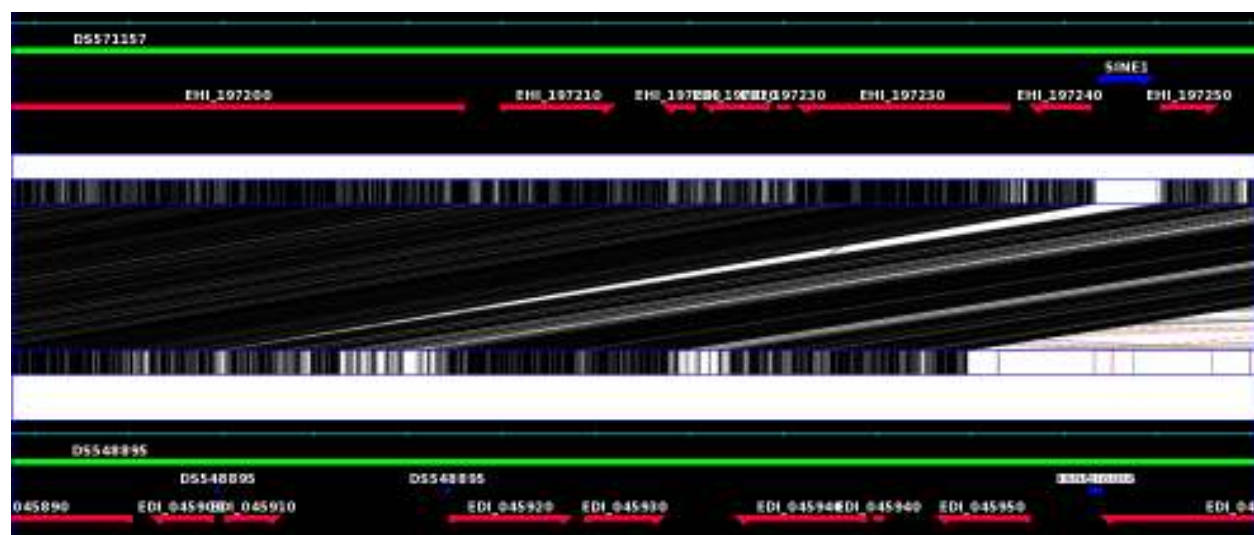

Figure. S2.

Supplement: Additional file 3 — Figure S2. Graphical representation of the syntenic region where SINE1 is present in E. histolytica but absent in E. dispar. For color code and arrows refer to figure S1-S17 [file 1471-2164-12-267-S3.PDF]

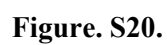

**Figure. S20.**

Supplement: Additional file 5 — Figure S20. Graphical representation of syntenic region where SINE1 is present in E. dispar but absent in E. histolytica. For color code and arrows refer to figure S1-S17. [file 1471-2164-12-267-S5.PDF]
