# Supplementary material for: Differential distribution of a SINE element in the Entamoeba histolytica and Entamoeba dispar genomes: Role of the LINE-encoded endonuclease
Source: BMC Genomics. 2011 May 25;12:267. doi: 10.1186/1471-2164-12-267 (PMC3118788; doi:10.1186/1471-2164-12-267)
Supplement: Additional file 2 — Table S1. Primer sets used for the construction of Ed EN. [file 1471-2164-12-267-S2.PDF]

Table S1 – Primer sets used for the construction of Ed EN.

No	5'<-----Sequence----->3'
1	T 7 promoter primer
2	TTTTTATACCCAC**T**AAATATTGC
3	GCAATATTT**A**GTGGGTATAAAAA
4	GATTTTTTATGAC**C**AATACAAGATAG
5	CTATCTTGTATT**G**GTCATAAAAAATC
6	CATATCATTGTGATGTC**A**TTATATTGGA
7	TCCAATATAA**T**GACATCACAATGATATGGAAC
8	TCTGGTCTTTTGTGGTAT**A**GTCCATCACTT
9	TGATGGAC**T**ATACCACAAAAGACCAGAC
10	CTTTAATTAGTCT**A**ATACTTTC**GGC**ATTTATTAATCC
11	GGATTAATAAAT**GCC**GAAAGTA**T**TAGACTAATTAAAG
12	GGTTTTTACTTCTTT**AGT**GATGTCAA
13	TTGACATC**ACT**AAAGAAGTAAAAAACC
14	TCGTCTTCATCTAGCAGCTCTAC**TAC**GTATGTTTGG
15	ATAC**GTA**GTAGAGCTGCTAGATGAAGACGAAGGG
16	GCGGCCGCATTAATAGAACTAGCTTCTATT**G**TTCCTGG
17	CATACGTAGTAGAGCTGC**AA**GATGAAGACGAAGGG
18	CCCTTCGTCTTCATC**TT**GCAGCTCTACTACGTATG
19	ATAGAA**A**C**A**AGTTCTATTAATACTTAAGCGGCCGCGCACCA
20	TGGTGCGCGGCCGCTTAAGTATTAATAGAACT**T**G**T**TTCTAT

Primer sets incorporating desired mutations (shown in bold) were designed from Eh EN sequence.
