# Supplementary material for: Differential distribution of a SINE element in the Entamoeba histolytica and Entamoeba dispar genomes: Role of the LINE-encoded endonuclease
Source: BMC Genomics. 2011 May 25;12:267. doi: 10.1186/1471-2164-12-267 (PMC3118788; doi:10.1186/1471-2164-12-267)
Supplement: Additional file 4 — Figure S3-S19. Graphical representation of the syntenic region where SINE1 is present in both E. histolytica and E. dispar. Top and bottom green lines are showing the genomes of E. histolytica and E. dispar respectively. Red arrows show the Eh and Ed genes, and the gene orientation. Shaded black or Red (connecting) lines show the identical region within E. histolytica and E. dispar. Darkness of the shade is proportional to the % Identity. Blue arrows show the repeat elements. SINE1 has been shown above the marked Yellow dot in both E. hitolytica and E. dispar in the syntenic region. [file 1471-2164-12-267-S4.PDF]

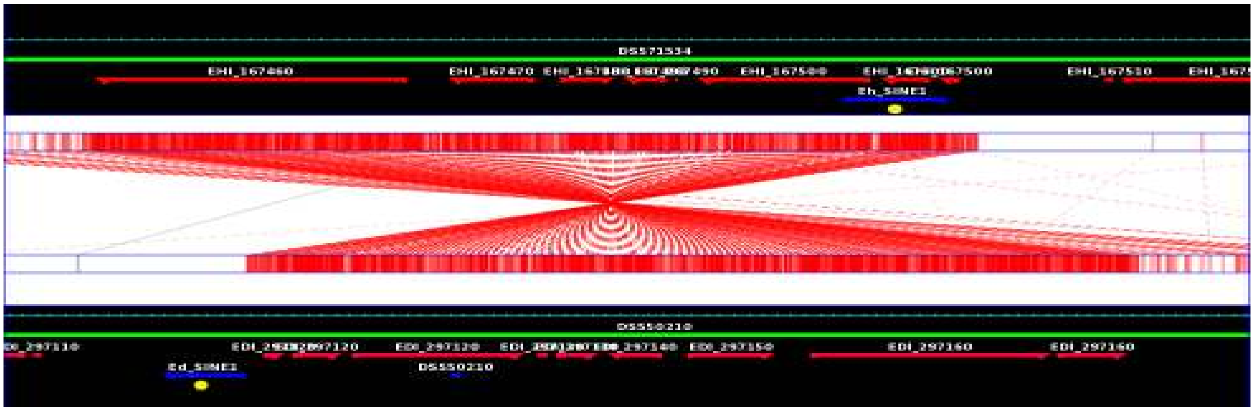

Figure. S3.

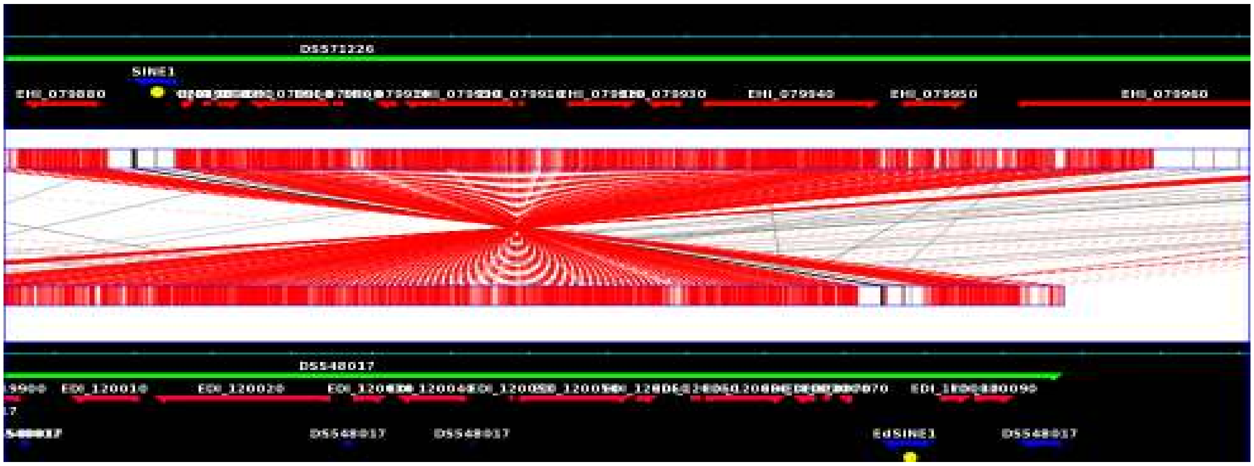

Figure. S4.

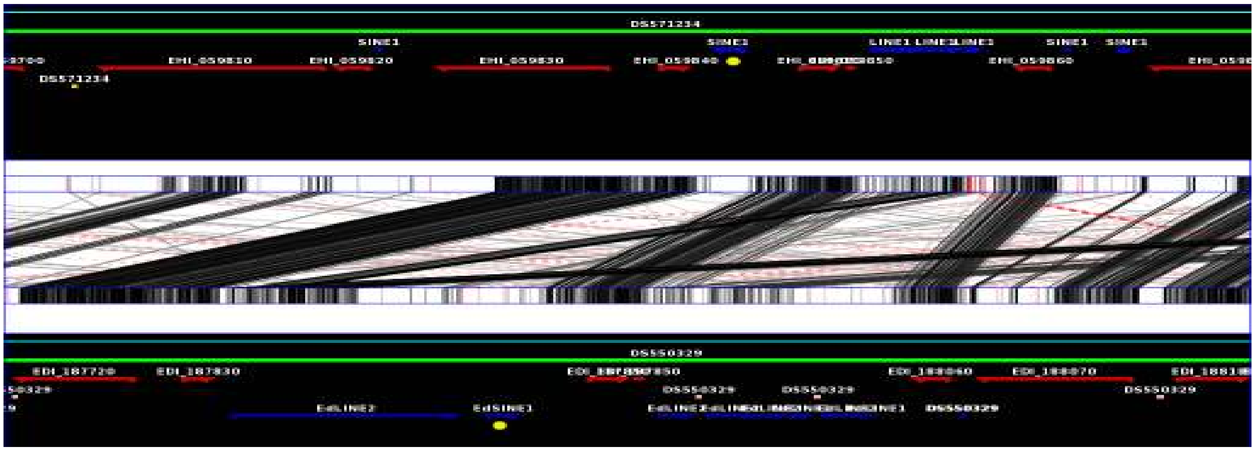

Figure. S5.

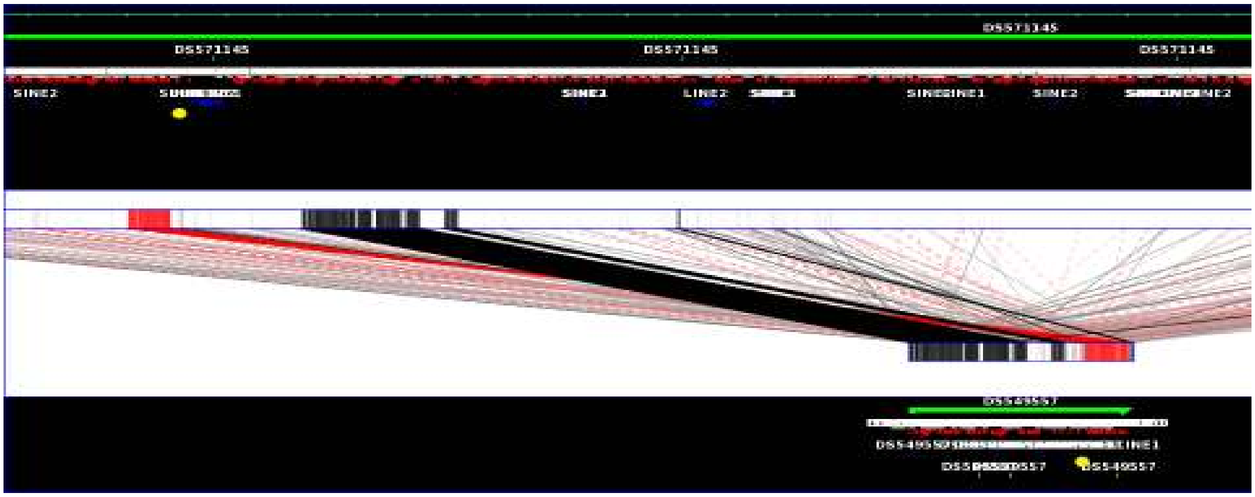

Figure. S6.

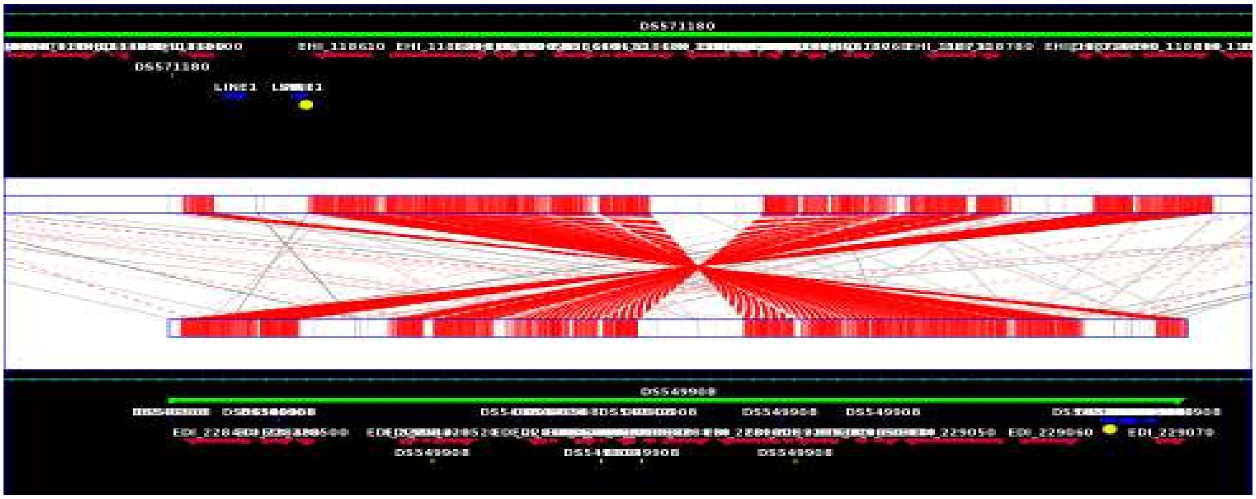

Figure. S7.

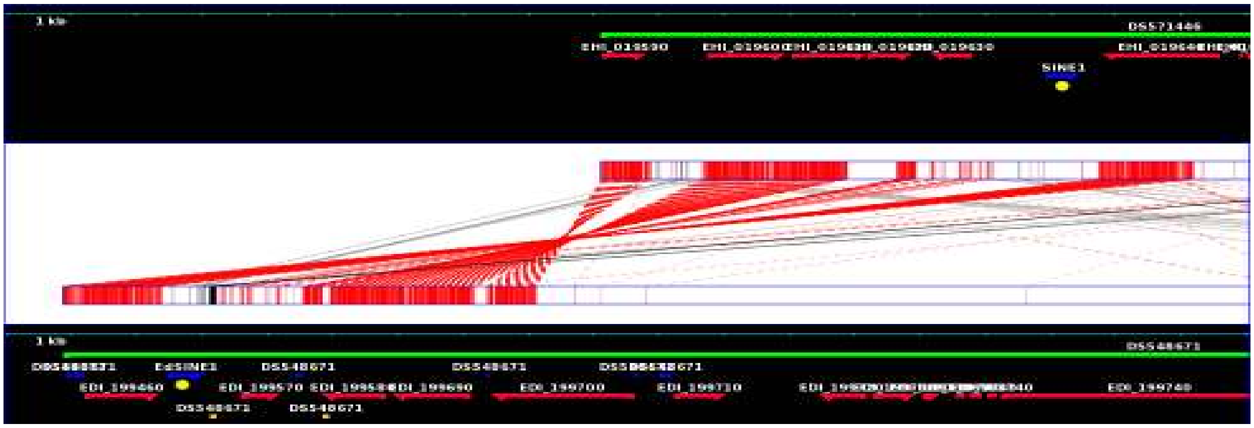

Figure. S8.

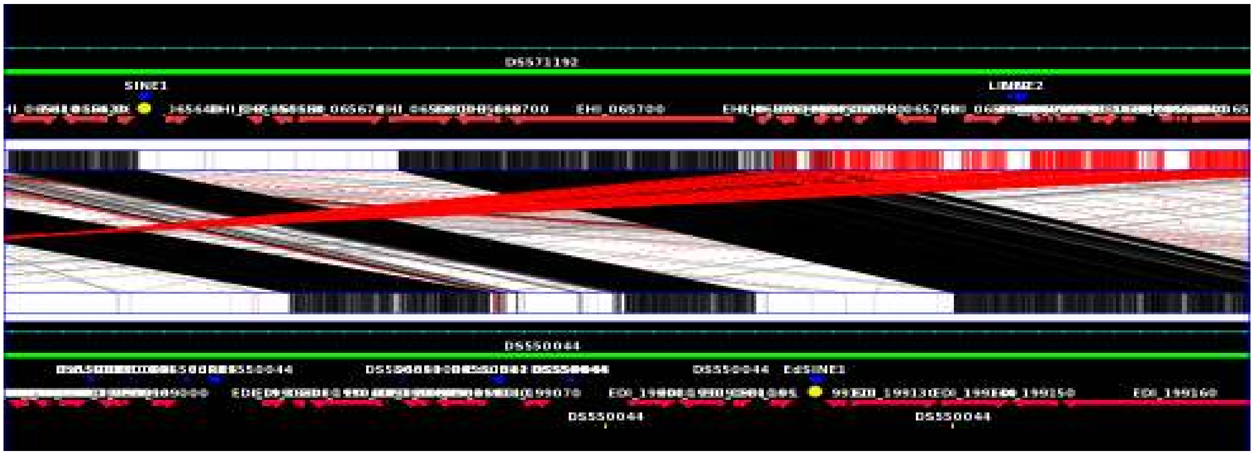

Figure. S9.

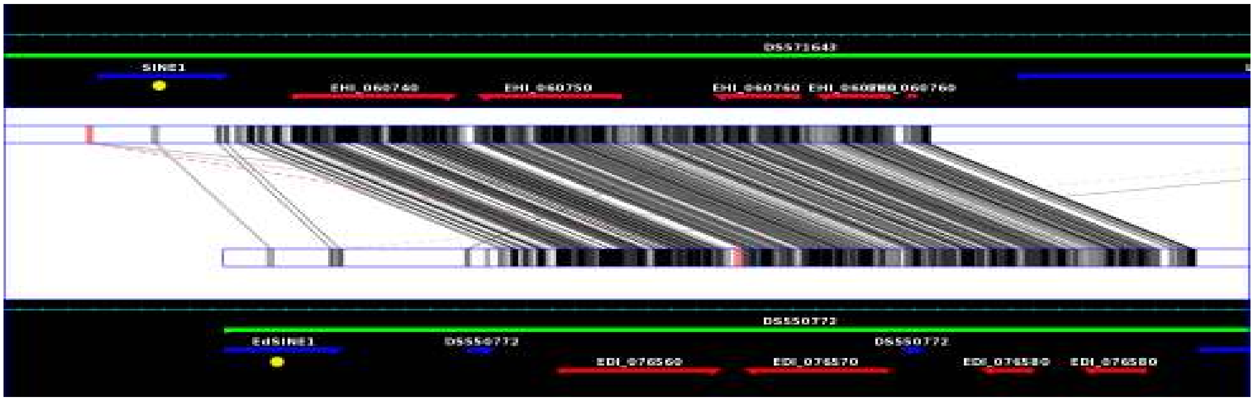

Figure. S10.

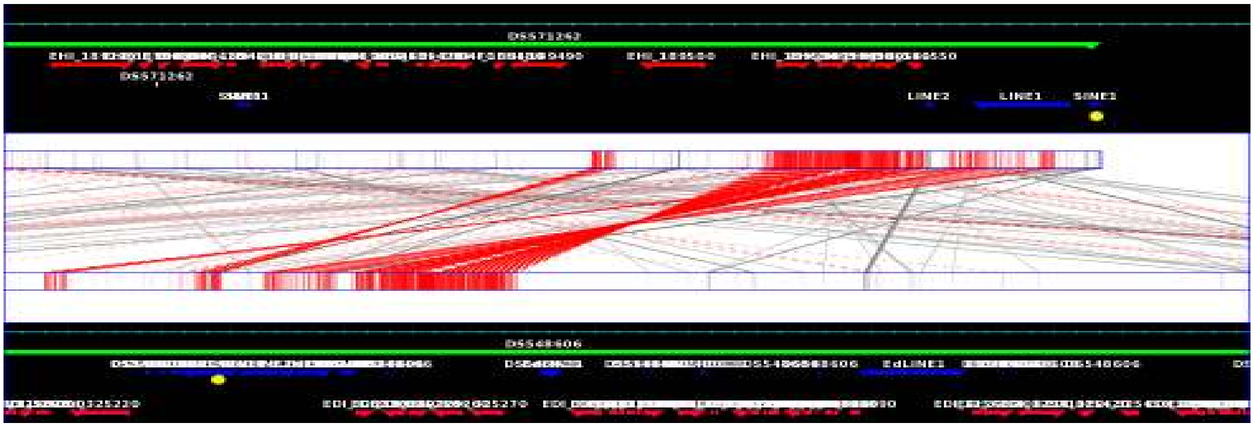

Figure. S11.

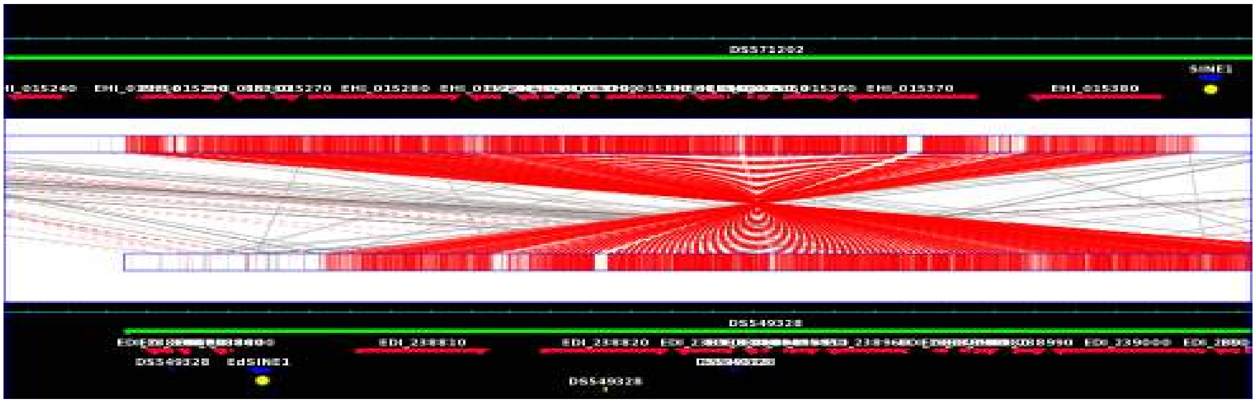

Figure. S12.

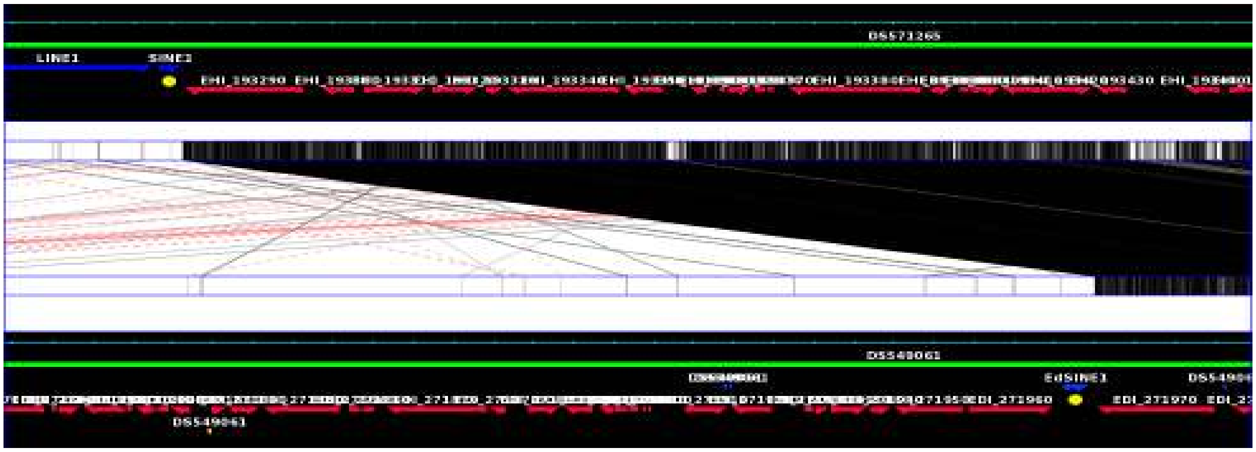

Figure. S13.

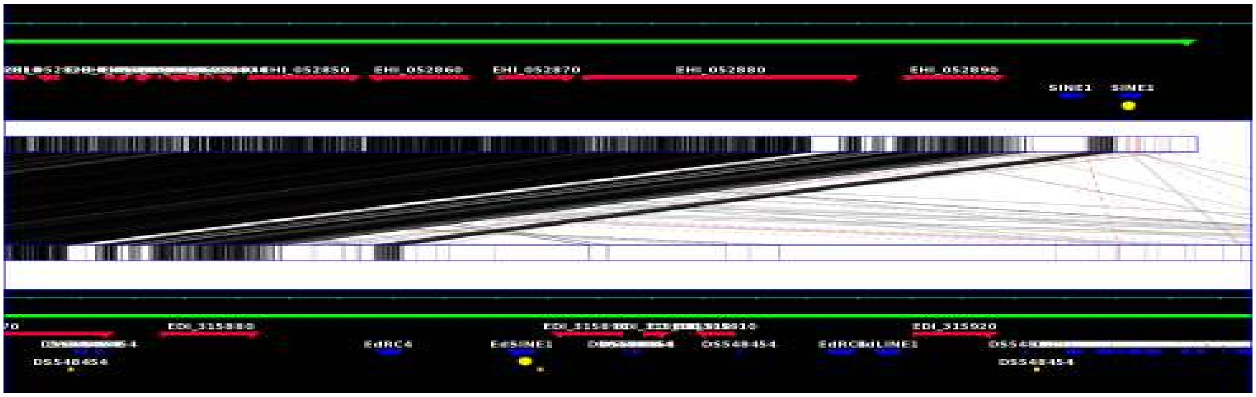

Figure. S14.

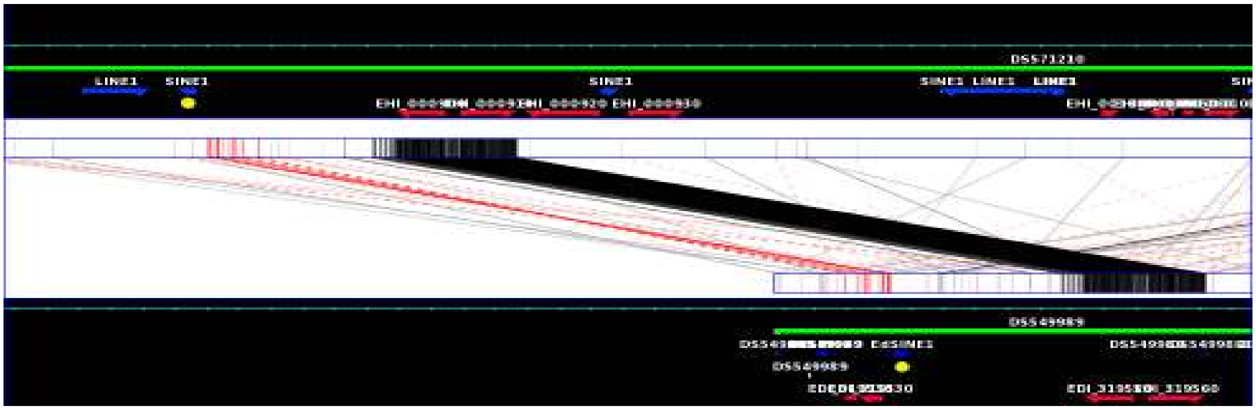

Figure. S15.

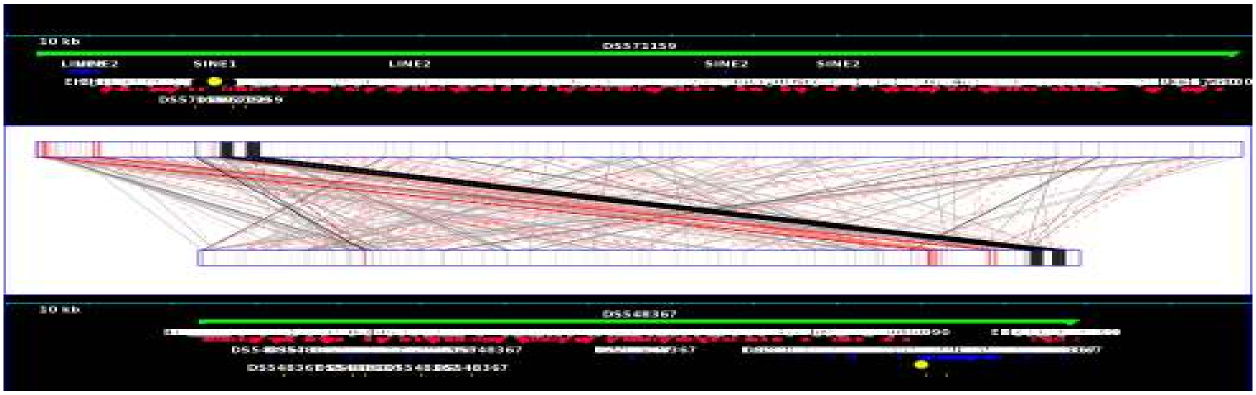

Figure. S16.

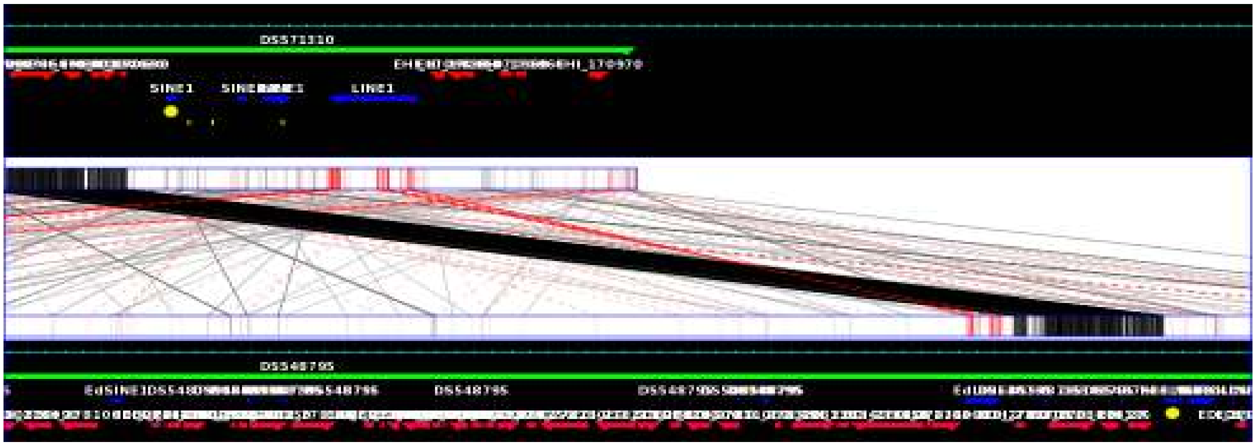

Figure. S17.

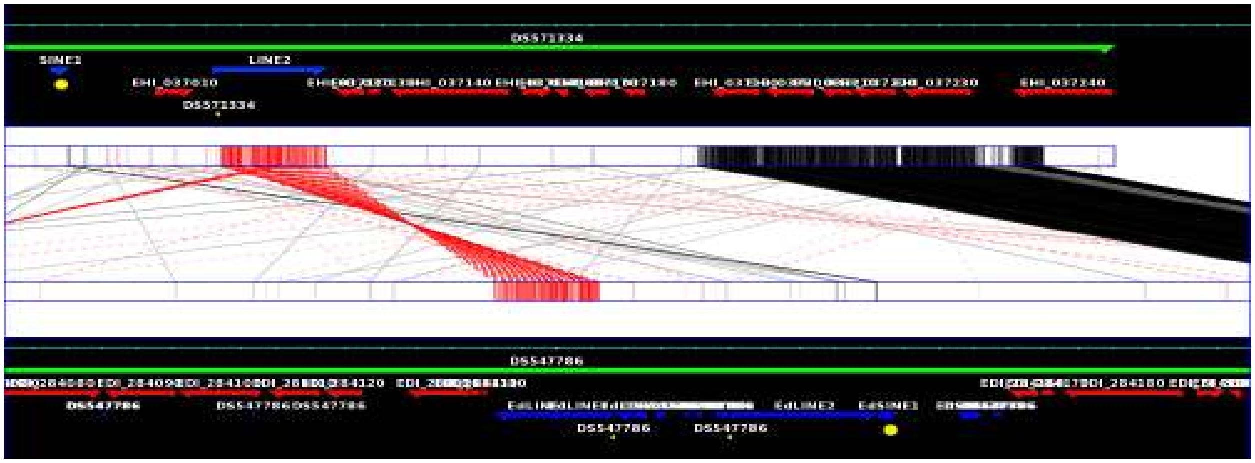

Figure. S18.

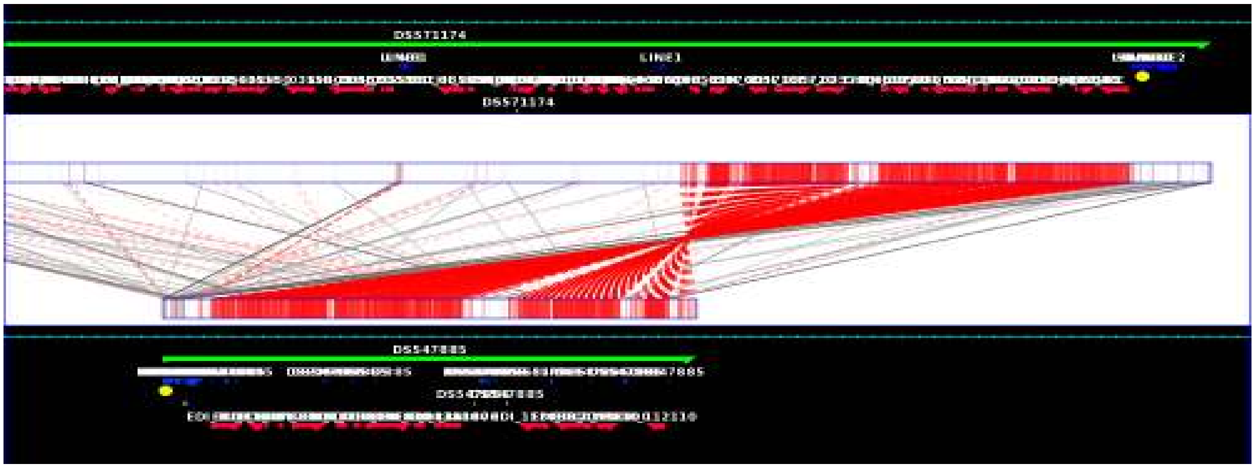

Figure. S19.
